# *MiR-150-3p* targets SP1 and suppresses the growth of glioma cells

**DOI:** 10.1042/BSR20180019

**Published:** 2018-05-15

**Authors:** Zhigang Tan, Jiaoying Jia, Yugang Jiang

**Affiliations:** Department of Neurosurgery, The Second Xiangya Hospital of Central South University (CSU), Changsha, Hunan 410011, China

**Keywords:** glioma, miR-150-3p, PTEN, SP1

## Abstract

Glioma has been considered as one of the most prevalent and common malignancy of the nervous system; however, the underlying mechanisms that are responsible for the occurrence and development of glioma still remain largely unknown. Amounting evidence highlights the critical regulatory function of miRNAs in carcinogenesis. Here, we showed that the expression of *miR-150-3p* was significantly decreased in glioma tissues and cell lines. Suppressed expression of *miR-150-3p* was associated with the lymph node metastasis of the glioma patients. Overexpression of *miR-150-3p* significantly inhibited the proliferation of glioma cells. Molecular study uncovered that the transcription factor specificity protein 1 (SP1) was identified as one of the targets of *miR-150-3p*. Highly expressed *miR-150-3p* in glioma cells significantly decreased both the mRNA and protein levels of SP1. Consistently, the abundance of phosphatase and tension homolog deleted on chromosome ten (PTEN), a negative downstream target of SP1, was increased with the ectopic *miR-150-3p*. Collectively, these results suggested that *miR-150-3p* suppressed the growth of glioma cells partially via regulating SP1 and possibly PTEN.

## Introduction

Glioma is the most common and malignant brain tumor, which accounts for approximately 80% of the brain carcinogenesis [1,2]. Surgical resection, radiotherapy, and chemotherapy have been the standard treatments for glioma. However, due to the high aggressive proliferation and invasion rate, as well as the resistance to necrosis, the medial survival of the glioma patients is only approximately 12 months and the 5-year survival rate of these patients remains less than 3% [3,4]. Therefore, it is quite urgent to identify novel targets and investigate the underlying molecular mechanisms which regulate the progression of glioma.

Increasing evidence has demonstrated the aberrant expression and critical involvement of miRNAs in human cancers [5–10]. MiRNAs were characterized as small (18–24 nts), single-stranded, non-coding RNA, which bind to the 3′-UTR of the target genes and inhibit the gene expression [11–13]. Due to the basic function of miRNAs in regulating gene expression, miRNAs are involved in a broad range of physiological processes, including cell proliferation, differentiation, stress condition, and tumorigenesis [13]. Notably, miRNA-mediated resistance to chemotherapy has been observed in a variety of cancers [14–19]. These studies indicated the critical roles of miRNAs in the initiation and progression of cancers. To search for miRNAs that were involved in the tumorigenesis of glioma, our previous work [20] screened the miRNAs that were aberrantly expressed in glioma tissues compared with normal brain tissues. The result showed that the expression of *miR-150-3p* was significantly decreased in glioma tissues (Supplementary Table S1), however, the potential function of *miR-150-3p* in glioma still remains unknown.

Specificity protein 1 (SP1) is a ubiquitously expressed transcription factor that recognizes the GC-boxes in the downstream target genes [21]. Overexpression of SP1 has been found in a variety of cancers and was correlated with the worse prognosis of the cancer patients [22–24]. Recent studies reported that the expression and activity of SP1 were regulated by miRNAs and affected the cancer progression [25–29]. Amongst these miRNAs, *miR-411* down-regulated the expression of SP1 and inhibited the growth of breast cancer cells [26]. Additionally, *miR-326* reversed chemoresistance in lung cancer by targetting SP1 [30]. It was also reported that *miR-31-5p* inhibited the proliferation, migration of HepG2 hepatocellular carcinoma (HCC) via targetting SP1 [31]. These studies demonstrated that SP1 served as a good target of miRNAs and involved in the tumorigenesis of human cancers. As a transcription factor, to understand the role of SP1 in cancers, the function of the downstream targets of SP1 deserves further investigation. Amongst these targets of SP1, the phosphatase and tension homolog deleted on chromosome ten (PTEN) is a well-established tumor suppressor, which is down-regulated or mutated in cancers and contributes to the initiation and development of cancers [32–34]. Recent study demonstrated that SP1 bound to the promoter of PTEN and negatively regulated the expression of PTEN [33]. This finding uncovered the novel molecular mechanism of SP1 in tumorigenesis via regulating PTEN.

In the present study, we detected the expression of *miR-150-3p* in glioma tissues and cell lines. The effect of *miR-150-3p* on the growth of glioma cells was investigated by overexpressing *miR-150-3p.* The downstream targets of *miR-150-3p* were predicted by the bioinformatics analysis and SP1 was predicted as one of the targets of *miR-150-3p*. The down-regulation of SP1 by *miR-150-3p* increased the expression of PTEN. Inverse correlation between the expression of *miR-150-3p* and SP1 was also observed in glioma tissues. These results uncovered the novel functional mechanism of *miR-150-3p* in glioma.

## Materials and methods

### Tissues and cell lines

Sixty glioma tissues were collected from the glioma patients by surgical reaction at The Second Xiangya Hospital of Central South University between January 2014 and October 2015. Thirty normal brain tissues were obtained from patients who underwent internal decompression surgery after traumatic brain injury. The tissues were immediately frozen in liquid nitrogen before miRNA extraction. The basic information of the patients including age, gender, lymph node metastasis, cancer stage and grade were summarized as Table 1. Written informed consent was obtained from all patients. The present study was approved by the Ethics Committee of The Second Xiangya Hospital of Central South University.

**Table 1:** Clinical characteristics of the glioma patients

Clinical parameters	*n*
**Age**	
<60	20
≥60	40
**Gender**	
Male	29
Female	31
**Cancer stage**	
I and II	22
III and IV	38
**Lymph node metastasis**	
Positive	39
Negative	21
**Tumor grade**	
G1–G2	18
G3	42

Human glioma cell lines including Uppsala 87 Malignant Glioma (U87-MG), U251, A172, SWO-38, and Suzhou Human Glioma-44 (SHG-44) were obtained from the Cell Bank of Chinese Academy of Sciences (Shanghai, China). These cells were cultured with Dulbecco’s modified Eagle’s medium (DMEM, Hyclone, UT, U.S.A.) supplemented with 10% FBS (Gibco, CA, U.S.A.). The normal human astrocytes cell NHA was purchased from Lonza (Basel, Switzerland) and cultured with the AGM™ BulletKit™ (Lonza, Basel, Switzerland), which contains basic medium, insulin, ascorbic acid, l-glutamine, rhEGF, GA-1000, and 10% FBS. All the cells were maintained at 37°C in a humidified atmosphere with 5% CO_2_.

### Oligonucleotides and cell transfection

The *miR-150-3p* mimics, mimics control miRNA, *miR-150-3p* antagomir, and antagomir negative control miRNA were chemically synthesized by RiboBio (Guangzhou, Guangdong, China). Cells were cultured with DMEM for 36 h and the transfection was performed with the Lipofectamine 2000 (Thermo Fisher Scientific, MA, U.S.A.) according to the manufacturer’s instructions. After transfection for 48 h, the expression level of *miR-150-3p* was determined by the real-time quantitative PCR (RT-qPCR) analysis.

### MiRNA isolation and quantitative real-time PCR

MiRNA extraction from the tissues or cell lines was performed with the miRcute miRNA isolation kit (DP501, Tiangen Biotechnology, Beijing, China). MiRNA was reverse transcribed with the miRcute miRNA First-Strand cDNA Synthesis Kit (KR201, Tiangen Biotechnology, Beijing, China) according to the manufacturer’s protocol. The qPCR reaction was performed with SsoFast™ EvaGreen® Supermix kit (Bio-Rad Laboratories, Inc., Hercules, CA, U.S.A.) on ABI Prism 7300 system (Applied Biosystems). The PCR conditions were set as follows: 95°C for 10 min and 40 cycles at 95°C for 15 s, 57°C for 1 min. The relative expression of *miR-150-3p* was normalized to the expression of U6 RNA with the 2^−ΔΔ*C*^_T_ method.

### Cell viability assay

Glioma cells transfected with *miR-150-3p* mimics or control miRNA were cultured in the 96-well plate. The cell viability was measured with cell counting kit-8 (CCK-8) at 0, 24, 48, 72, and 96 h after transfection. Ten microliters of CCK-8 reagent was added into the cells and incubated at 37°C for 2 h. The absorbance of each well was determined at 450 nm with the microplate reader. The experiment was performed in triplicate.

### Colony formation assay

Glioma cells with overexpressed *miR-150-3p* or control miRNA were seeded into the six-well plate with a density of 500 cells/well. Cells were cultured for 2 weeks with fresh medium containing 10% FBS. To observe the formation of colonies, cell culture medium was discarded and the cells were washed with PBS for three times. And then cells were fixed with 100% methanol for 30 min at room temperature (RT) and stained with 0.1% Crystal Violet for 20 min. The colonies were observed with the microscope and the number of colonies was recorded.

### Western blot

After transfection for 48 h, glioma cells were harvested and lysed with the NP-40 lysis buffer (150 mM NaCl, 1% NP-40, 50 mM Tris/HCl (pH 8.0), 1 mM EDTA) on ice for 30 min in the presence of protease inhibitor. The protein concentration was quantitated with the BCA Protein Assay Kit (Beyotime Biotechnology, Shanghai, China). Twenty micrograms protein of each sample was loaded and separated by the SDS/PAGE (15% gel). The protein was then transferred on to the nitrocellulose membrane (Millipore, Billerica, MA, U.S.A.) and blocked with 5% of non-fat milk at RT for 1 h. Subsequently, membrane was incubated with primary antibodies against PTEN (ab32199, 1:2000 dilution, Abcam), GAPDH (sc-20357, 1:3000 dilution, Santa Cruz Biotechnology, Inc., Dallas, TX, U.S.A.), SP1 (#5931, 1:2000 dilution, Cell Signaling Technology, Danvers, MA, U.S.A.) for 2 h at RT, respectively. After washing with TBS and Tween-20 (TBST), the membrane was incubated with the secondary antibody conjugated with horseradish peroxidase (HRP) for 1 h at RT. The protein bands were detected with ECL (Amersham Pharmacia Biotech, Little Chalfont, U.K.) and analyzed using ImageJ Software (version 1.62; National Institute of Health, Bethesda, MD, U.S.A.).

### Dual-luciferase report assay

The wild-type (WT) or mutant 3′-UTR of SP1 was synthesized and constructed into the pGL3 luciferase reporter vector (Promega Corporation, Madison, WI, U.S.A.). Glioma cells were cultured in the 96-well plate and co-transfected with WT or mutant pGL3-SP1-3′-UTR in the presence of *miR-150-3p* mimics. The pGL3 *Renilla* luciferase reporter plasmid was transfected as the internal control. After transfection for 48 h, cells were harvested and the luciferase activity was measured using the Dual-luciferase Reporter Assay Kit (Promega Corporation, Madison, WI, U.S.A.). The experiment was performed in triplicate.

### Cell apoptosis

The percentage of cell apoptosis was determined with the Annexin V-FITC Apoptosis Detection kit (Thermo Fisher Scientific, MA, U.S.A.) according to the manufacturer’s instructions. Glioma cells were transfected with *miR-150-3p* mimics or control miRNA. After transfection for 48 h, cells were harvested, washed, and resuspended to approximately 1 × 10^6^ cells/ml with the binding buffer. And then 5 µl annexin V and 1 µl of 100 µg/ml propidium iodide (PI) were added into 100 µl of the cell suspension and incubated at RT for 15 min. Finally, 400 µl binding buffer was added and the stained cells were analyzed using the Beckman Coulter Epic XL flow cytometer.

### Statistical analysis

The data were presented as mean ± S.D. All statistical analyses were performed with GraphPad Prism version 6 (GraphPad Prism version 6.0, Inc., California, USA). The differences between two or more groups were analyzed by Student’s *t* test or one-way ANOVA followed by Bonferroni’s multiple comparison tests. *P*<0.05 was considered as significant.

## Results

### *miR-150-3p* was down-regulated in glioma tissues and cell lines

**Figure 1:**
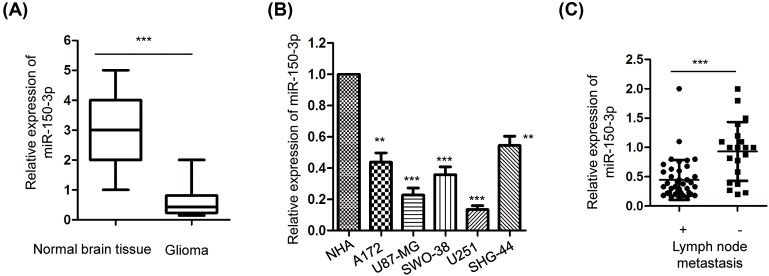
*miR-150-3p* was significantly decreased in glioma tissues and cell lines (**A**) RT-qPCR analysis of the expression level of *miR-150-3p* in glioma tissues (*n*=60) and normal tissues (*n*=30). U6 RNA was used as the internal control. ***P<0.001, Student’s *t* test. Data were obtained from three technical replicates. (**B**) Relative expression of *miR-150-3p* in NHA and glioma cell lines. **P<0.01, ***P<0.001, one-way ANOVA test. Data were obtained from three technical replicates. (**C**) Comparison of the expression of *miR-150-3p* in glioma patients with positive (*n*=20) or negative (*n*=40) lymphoma node metastasis. ***P<0.001, Student’s *t* test. Data were obtained from three technical replicates.

To detect the expression of *miR-150-3p* in glioma, RT-qPCR analysis was performed with glioma tissues and normal brain tissues. As shown in Figure 1A, the expression of *miR-150-3p* was significantly decreased in glioma tissues in comparison with that of the normal brain tissue. Consistently, the expression of *miR-150-3p* in glioma cell lines including U87-MG, U251, A172, SWO-38, and SHG-44 was measured and the data showed that the level of *miR-150-3p* was significantly decreased in all the above glioma cell lines compared with that of the normal astrocytes cells NHA (Figure 1B).

To further characterize the relationship between the expression of *miR-150-3p* and the progression of glioma, the association between the expression abundance of *miR-150-3p* and the lymph node metastasis of the glioma patients was analyzed. The data showed that compared with patients without lymph node metastasis, significantly decreased expression of *miR-150-3p* was observed in the glioma tissues from patients bearing lymph node metastasis (Figure 1C). These results indicated the down-regulation of *miR-150-3p* in glioma tissues and might be correlated with the metastasis of glioma.

### Overexpression of *miR-150-3p* inhibited the proliferation of glioma cells

**Figure 2:**
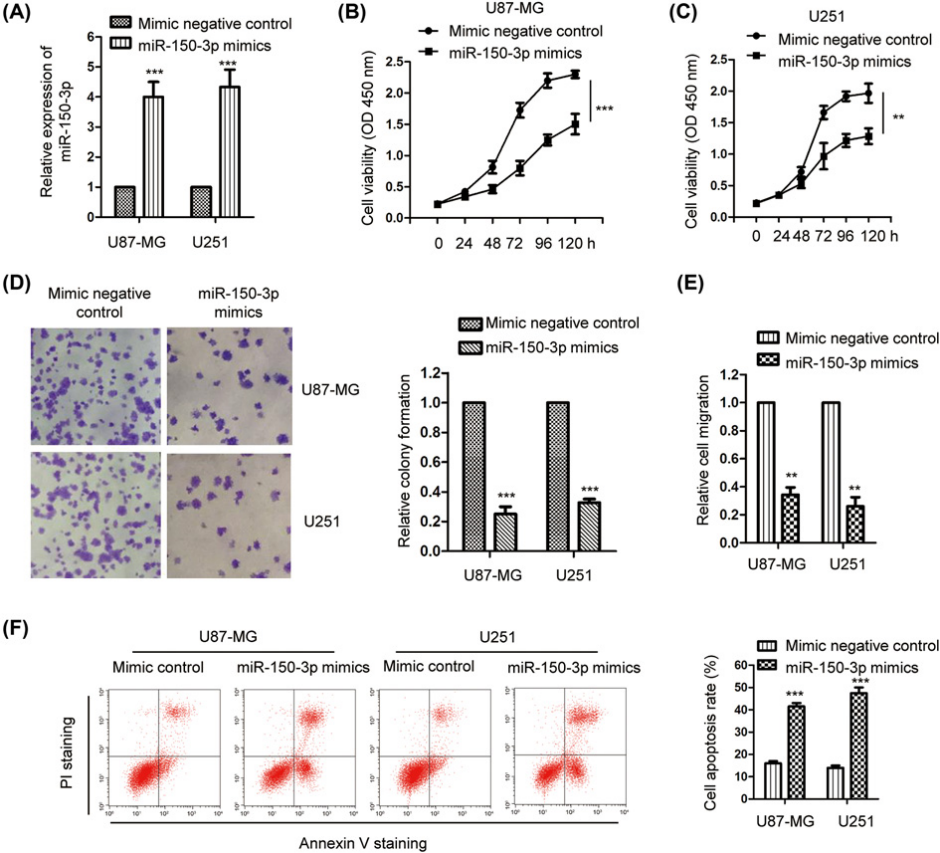
Overexpression of *miR-150-3p* inhibited the growth of glioma cells (**A**) The level of *miR-150-3p* in both U87-MG and U251 cells that were transfected with *miR-150-3p* mimics or control miRNA were detected by RT-qPCR. ***P<0.001, Student’s *t* test. Data were obtained from three biological replicates. (**B**,**C**) Effect of *miR-150-3p* on the viability of glioma cells was determined by the CCK-8 assay. **P<0.01, ***P<0.001, two-way ANOVA test. Data were obtained from three biological replicates. (**D**) Colony formation of both U87-MG and U251 cells that harbored *miR-150-3p* mimics was significantly decreased. ***P<0.001, Student’s *t* test. Experiment was performed with three biological replicates. (**E**) Glioma cells were transfected with *miR-150-3p* mimics or control vector, and the cell migration of the cells were compared. **P<0.01, Student’s *t* test. Experiment was performed with three biological replicates. (**F**) The percentage of cell apoptosis of glioma cells expressing *miR-150-3p* mimics or control miRNA was determined. ***P<0.001, Student’s *t* test. Data were obtained from three biological replicates.

Due to the aberrant expression of *miR-150-3p* in glioma, to detect the effect of *miR-150-3p* on the growth of glioma cells, U87-MG and U251 cells harboring relatively low abundance of *miR-150-3p* amongst the glioma cell lines we used, were transfected with *miR-150-3p* mimics or control miRNA. The expression level of *miR-150-3p* was confirmed by RT-qPCR (Figure 2A). The results showed that overexpressed *miR-150-3p* significantly inhibited the viability of both U87-MG and U251 cells as detected by the CCK-8 assay (Figure 2B,C). To confirm the inhibition of *miR-150-3p* on the growth of glioma cells, colony formation of both U87-MG and U251 cells that transfected with highly expressed *miR-150-3p* was evaluated. The results indicated that compared with the control group, overexpression of *miR-150-3p* significantly decreased the colony formation of glioma cells (Figure 2D). Consistent with these data, cells harboring highly expressed *miR-150-3p* presented decreased cell migration ability in comparison with that of cells expressing control miRNA (Figure 2E). To further validate the influence of *miR-150-3p* on the growth of glioma cells, the cell apoptosis rate of both U87-MG and U251 cells transfected with *miR-150-3p* mimics or control miRNA was detected. As shown in Figure 2F, overexpression of *miR-150-3p* significantly enhanced the apoptosis rate of both U87-MG and U251 cells. These data demonstrated the negative regulation of *miR-150-3p* on the growth of glioma cells.

### SP1 was a target of *miR-150-3p* in glioma cells

**Figure 3:**
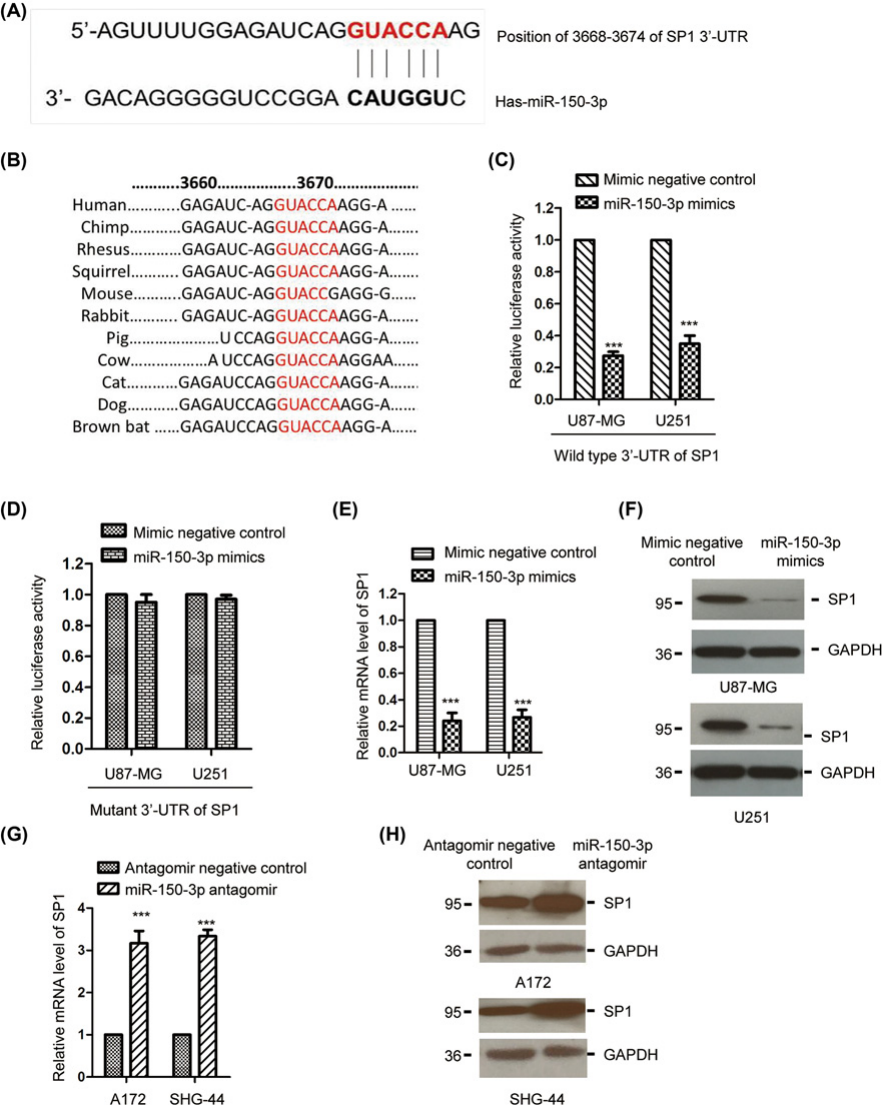
SP1 was a downstream target of *miR-150-3p* (**A**) The putative binding site of *miR-150-3p* in the 3′-UTR of SP1. (**B**) The predicted binding sites of *miR-150-3p* in the 3′-UTR of SP1 were highly conserved amongst different species. (**C**,**D**) The effect of *miR-150-3p* on the luciferase intensity of WT or mutant 3′-UTR of SP1 was determined by the dual-luciferase activity. ***P<0.001, Student’s *t* test. Data were obtained from three biological replicates. (B) Both U87-MG and U251 cells were transfected with *miR-150-3p* mimics or control miRNA, and the mRNA level of SP1 was detected. ***P<0.001, Student’s *t* test. Data were obtained from three biological replicates. (**F**) Glioma cells transfected with control miRNA or *miR-150-3p* mimics were collected and the protein abundance of SP1 was determined by Western blot with anti-SP1 antibody. Experiment was performed with three biological replicates. (**G**,**H**) The endogenous *miR-150-3p* was down-regulated in A172 and SHG-44 cells. The mRNA and protein level of SP1 was detected by RT-qPCR and Western blot, respectively. Experiment was performed with three biological replicates.

To understand the underlying mechanism by which *miR-150-3p* regulated the growth of glioma cells, the downstream targets of *miR-150-3p* were predicted by the TargetScan database. Amongst all the candidates, the 3′-UTR of SP1 was found to have the putative binding site of *miR-150-3p* (Figure 3A). Furthermore, the predicted binding site of *miR-150-3p* in the 3′-UTR of SP1 was highly conserved across different species including human, chimp, rhesus, squirrel, rabbit, pig, cow, cat, dog, and brown bat) (Figure 3B). To confirm the interaction between *miR-150-3p* with the 3′-UTR of SP1, luciferase reporter assay was performed by co-transfecting the plasmid containing WT or mutant 3′-UTR of SP1 in the presence of *miR-150-3p* mimics or control miRNA. As shown in Figure 3C,D, compared with the control cells, overexpression of *miR-150-3p* significantly decreased the luciferase activity of vector bearing WT but not mutant 3′-UTR of SP1 in both U87-MG and U251 cells.

To further explore the effect of *miR-150-3p* on the expression of SP1, glioma cells were transfected with *miR-150-3p* mimics or control miRNA and the mRNA level of SP1 was detected. As shown in Figure 3E, transfection of *miR-150-3p* significantly decreased the mRNA abundance of SP1. Consistently, the protein level of SP1 was also examined by Western blot and reduced protein expression of SP1 was observed in both U87-MG and U251 cells that bore overexpressed *miR-150-3p* (Figure 3F). To provide more evidence to characterize the negative regulation between *miR-150-3p* and SP1, the endogenous *miR-150-3p* was down-regulated by transfecting *miR-150-3p* antagomir into the glioma cells A172 and SHG-44, which harbored relatively higher expression of *miR-150-3p* amongst all the glioma cells we used. As presented in Figure 3G,H, both the mRNA and protein level of SP1 of glioma cells were increased with the reduction in *miR-150-3p*. These data demonstrated that *miR-150-3p* targetted SP1 and negatively regulated the expression of SP1 in glioma cells.

### Overexpression of *miR-150-3p* negatively regulated the SP1- PTEN pathway

**Figure 4:**
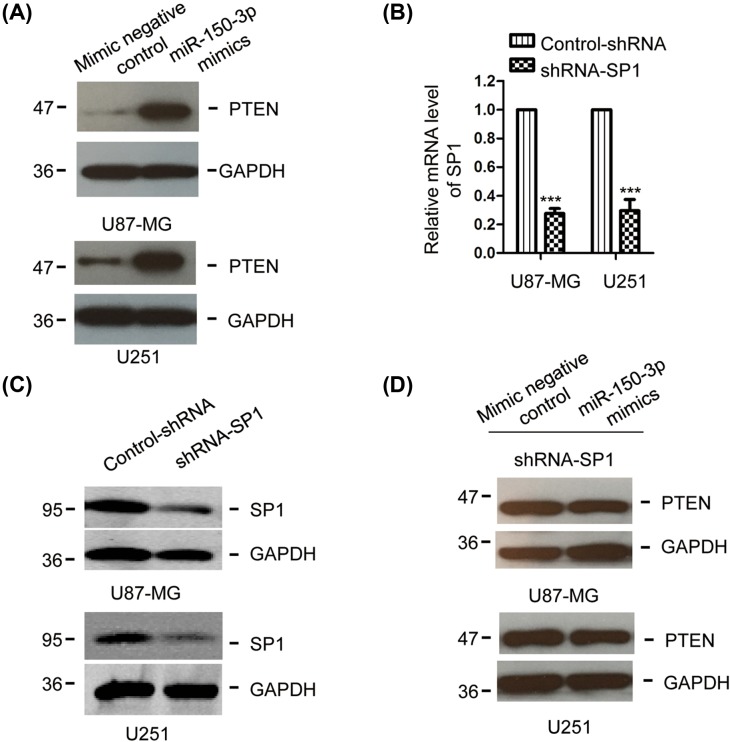
*miR-150-3p* regulated the SP1-PTEN pathway (**A**) Glioma cells were transfected with *miR-150-3p* mimics or control vector and the protein level of PTEN was detected by Western blot with anti-PTEN antibody. Experiment was performed with three biological replicates. (**B**,**C**) The endogenous SP1 was down-regulated by transfecting control-shRNA or shRNA-SP1. The depletion of SP1 was confirmed by RT-qPCR and Western blot. Experiment was performed with three biological replicates. (**D**) *MiR-150-3p* or control miRNA was transfected into glioma cells with depleted SP1, and the protein abundance of PTEN was detected. Experiment was performed with three biological replicates.

Previous studies demonstrated that SP1 promoted the cancer cell migration and proliferation via inhibiting PTEN [33,35]. As *miR-150-3p* decreased the expression of SP1, to detect whether *miR-150-3p* regulates the expression of PTEN, glioma cells were transfected with *miR-150-3p* mimics or control miRNA, and the protein abundance of PTEN was examined by Western blot. As shown in Figure 4A, overexpression of *miR-150-3p* promoted the expression of PTEN in both U87-MG and U251 cells. To confirm the regulation of *miR-150-3p* on PTEN through SP1, the endogenous expression of SP1 was depleted by shRNA-SP1, the down-regulation efficiency of SP1 was confirmed by RT-qPCR and Western blot analyses (Figure 4B,C). U87-MG and U251 cells bearing depleted SP1 were transfected with *miR-150-3p* mimics or control miRNA and the protein expression of PTEN was detected. As shown in Figure 4D, highly expressed *miR-150-3p* in SP1 depleted cells failed to promote the expression of PTEN. These results suggested that *miR-150-3p* negatively regulated the SP1-PTEN pathway.

### *miR-150-3p* was negatively correlated with the expression level of SP1 in glioma patients

**Figure 5:**
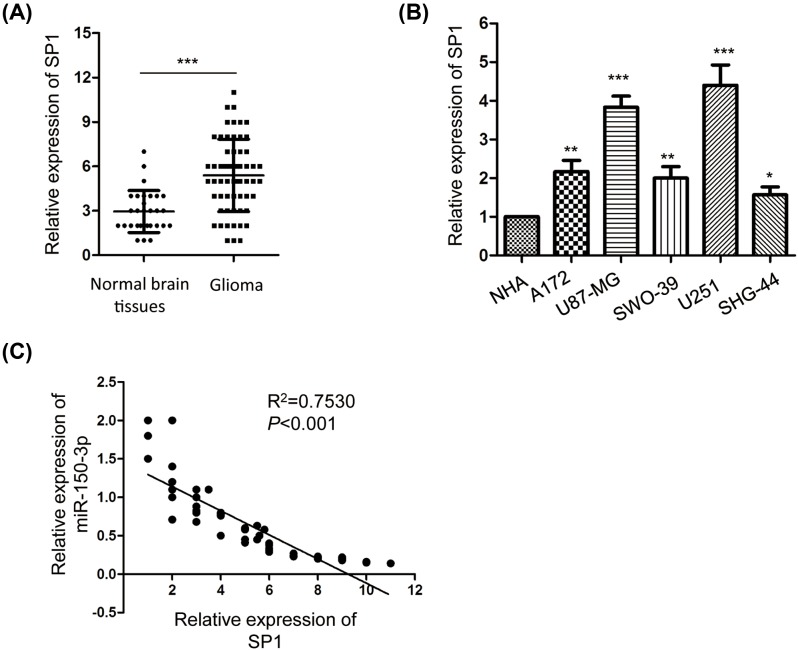
SP1 was highly expressed in glioma tissues and inversely correlated with the expression of *miR-150-3p* (**A**,**B**) The mRNA level of SP1 in glioma tissues or cell lines was examined by RT-qPCR analysis. ***P<0.001, Student’s *t* test. Data were obtained from three biological replicates. (**C**) The correlation between the expression of *miR-150-3p* and SP1 in glioma tissues was analyzed. *P<0.05,**P<0.01

As SP1 was identified as one of the downstream targets of *miR-150-3p* and aberrant expression of SP1 had been found to be associated with the development of cancer, we analyzed the expression level of SP1 in glioma tissues. The result showed that the expression of SP1 was significantly up-regulated in glioma tissues compared with that of the normal tissues (Figure 5A). Consistently, the expression level of SP1 in glioma cells including A172, U87-MG, SWO-38, U251, and SHG-44 was also notably increased in comparison with that of the normal cell NHA (Figure 5B). Furthermore, the correlation between the expression of SP1 and *miR-150-3p* in glioma tissues was also analyzed. The data indicated that the expression of *miR-150-3p* was significantly inversely correlated with the level of SP1 (Figure 5C). These results suggested the negative correlation between *miR-150-3p* and SP1 in glioma tissues.

## Discussion

In recent years, the function of miRNAs is widely explored in tumorigenesis. In the present study, we demonstrated that *miR-150-3p* was down-regulated in glioma tissues and cell lines. Overexpression of *miR-150-3p* inhibited the growth of glioma cells. The underlying molecular mechanism found that *miR-150-3p* targetted and down-regulated the expression of SP1. These findings provided the possible functional mechanism of *miR-150-3p* in glioma.

Recent analyses of miRNA expression by RNA sequencing demonstrated that *miR-150-3p* was significantly down-regulated in head and neck squamous cell carcinoma and acted as an antitumor miRNA in HNSCC [31]. In HCC, *miR-150-3p* was identified as oxidative response miRNA and regulated the oxidative stress-related gene expression [36]. Kaplan–Meier analysis showed that *miR-150-3p* was significantly associated with the overall survival of HCC patients [36]. Consistent with these findings, in this study, decreased expression of *miR-150-3p* was observed in glioma tissues, which was associated with the lymph node metastasis of the glioma patients. Further functional study of *miR-150-3p* demonstrated that overexpression of *miR-150-3p* significantly inhibited the viability, migration, and colony formation of glioma cells. These results suggested the inhibitory effect of *miR-150-3p* on the growth of glioma cells, and the involvement of *miR-150-3p* in other types of cancers deserves further investigation.

The regulatory mechanism of miRNAs was achieved through inhibiting the expression of downstream target genes. With the bioinformatics analysis, SP1 was predicted as one of the targets of *miR-150-3p*. This observation was supported by the results that *miR-150-3p* decreased the luciferase activity of the 3′-UTR of SP1 and negatively regulated both the mRNA and protein expression of SP1. It has been documented that SP1 was a basal transcription factor in recruiting the general transcription machinery [21]. SP1 was overexpressed in a variety of human cancers and regulated the expression of genes involved in cell proliferation, differentiation, apoptosis, and angiogenesis [21,37–39]. Besides, highly expressed SP1 was associated with the poor prognosis in cancer patients [39]. Interestingly, SP1 has been the target of miRNAs in many types of cancers [25–28]. For example, *miR-760* inhibits the tumorigenesis of colon cancer via regulating SP1 [40]. Increased expression of *miR-31-5p* inhibited the expression of SP1 and suppressed the proliferation of HCC [41]. Additionally, *miR-376a* was found to inhibit the growth of glioblastoma multiforme via targetting SP1 [42]. A recent study reported that *miR-377* inhibited the proliferation and invasion of glioma cells though directly targetting SP1 [43]. Combined with our results, SP1 was a promising target of different miRNAs in glioma that might function together to inhibit the carcinogenesis of glioma. As one of the downstream targets of SP1, overexpression of *miR-150-3p* increased the expression abundance of PTEN. These results demonstrated that *miR-150-3p* suppressed the glioma cell growth in part by SP1 and possibly PTEN. Due to the multiple targets of miRNAs, in addition to SP1, searching for other downstream targets of *miR-150-3p* and related pathways that might partially mediate the role of *miR-150-3p* in glioma cells is also an interesting topic to provide novel insights into the function of *miR-150-3p* in glioma.

In conclusion, the results of the present study uncovered the decreased expression of *miR-150-3p* in glioma tissues and cell lines. Highly expressed *miR-150-3p* suppressed the growth of glioma cells partially via targetting the SP1-PTEN signaling pathway. These findings provided novel insights into the functional mechanism of *miR-150-3p* in glioma.

## Supporting information

**Supplementary Table 1:** miRNA expression profile in glioma tissues compared with that of the paired normal tissue.

## Abbreviations

CCK-8: cell counting kit-8
DMEM: Dulbecco’s modified Eagle’s medium
GAPDH: glyceraldeyde-3-phosphate dehydrogenase
GA-100: gentamycin-amphotericin B mix
HCC: hepatocellular carcinoma
HNSCC: head and neck squamous carcinoma
PTEN: phosphatase and tension homolog deleted on chromosome ten
qPCR: quantitative PCR
rhEGF: recombinant human epidermal growth factor
RT: room temperature
RT-qPCR: real-time quantitative PCR
SHG-44: Suzhou human glioma-44
SP1: specificity protein 1
U87-MG: uppsala 87 malignant glioma
WT: wild-type
