# Supplementary material for: MiR-150-3p targets SP1 and suppresses the growth of glioma cells
Source: Biosci Rep. 2018 May 15;38(3):BSR20180019. doi: 10.1042/BSR20180019 (PMC6048207; doi:10.1042/BSR20180019)
Supplement: Supplementary file 1 [file bsr20180019_Supp1.pdf]

Supplementary Table 1. miRNA expression profile in glioma tissues compared with that of the paired normal tissue.

**miRNA**	**Fold change**	***P* value**	**Regulation**
miR-373	14.82	****	Up
miR-487a	11.42	**	Up
miR-362-5p	8.44	***	Up
miR-19a	6.96	****	Up
miR-944	4.92	**	Up
miR-200	2.82	**	Up
miR-762	2.24	**	Up
miR-544a	1.62	**	Up
miR-221	1.44	**	Up
miR-711	1.28	***	Up
miR-150-3p	0.08	****	Down
miR-497	0.12	****	Down
miR-379	0.20	****	Down
miR-148a	0.24	****	Down
miR-328-3p	0.36	***	Down
miR-215	0.40	**	Down
miR-30a	0.44	**	Down
miR-133	0.46	**	Down
miR-375	0.46	**	Down
miR-101	0.50	**	Down

**$P<0.01$, ***$P<0.001$, ****$P<0.0001$

Fold change, the expression miRNA in glioma tissues vs. adjacent normal tissue.
